# Retraction: Multidisciplinary Surgical Management of Concurrent Traumatic Brain and Abdominal Injuries: A Systematic Review of Integrated Care Approaches and Clinical Outcomes

**DOI:** 10.7759/cureus.r196

**Published:** 2025-10-17

**Authors:** David O Oriko, Jarallah H. J. Alkhazendar, Aliaa H Alkhazendar, Mawada Taha, Dr Abdul Sattar Gatta, Asma Ahmed Osman Mohamed, Hussein Taha, Rana Muhammad Haris, Shahzad Ahmad, Manahil Awan

**Affiliations:** 1 Neurology, University of Maryland Medical Center, Baltimore, USA; 2 General and Emergency Surgery, East and North Hertfordshire NHS Trust-Lister Hospital, Stevenage, GBR; 3 Surgery, Islamic University of Gaza, Gaza, PSE; 4 General Surgery, National Ribat University, Khartoum, SDN; 5 Accident and Emergency, Liaquat National Hospital, Karachi, PAK; 6 Medicine, Liaquat College of Medicine and Dentistry, Karachi, PAK; 7 Surgery, Burjeel Medical City, Burjeel, ARE; 8 Surgery, Nahda College, Khartoum, SDN; 9 General Surgery, Doctors Hospital and Medical Center, Lahore, PAK; 10 Surgery, Liaquat National Hospital, Karachi, PAK; 11 General Practice, Executive and Special Ward, Liaquat National Hospital, Karachi, PAK

The Editors-in-Chief have retracted this article. An investigation by the journal found evidence of authorship manipulation. The Editors-in-Chief therefore no longer have confidence in the provenance and reliability of the article contents. The authors disagree with the decision to retract.

